# Prevalence of transfusion-transmitted infections in Saudi Arabia blood donors: A nationwide, cross-sectional study

**DOI:** 10.15537/smj.2022.43.12.20220634

**Published:** 2022-12

**Authors:** Jawaher Alsughayyir, Yasser Almalki, Imtinan Alburayk, Mohrah Alalshaik, Ibrahim Aljoni, Mona Kandel, Mohammad A. Alfhili, Abdulmajeed A. Alabdullateef

**Affiliations:** *From the Chair of Medical and Molecular Genetics Research (Alfhili), Department of Clinical Laboratory Sciences, and from the Department of Clinical Laboratory Sciences, (Alsughayyir, Alalshaik,), College of Applied Medical Sciences, King Saud University; from the Division of Hematology (Almalki), King Khalid University Hospital; from the Blood Transfusion Services Operation Centre (Aljoni, Kandel, Alabdullateef Ministry of Health, Riyadh; and from the Maternity and Children Hospital (Alburayk), Ministry of Health, Hail, Kingdom of Saudi Arabia.*

**Keywords:** transfusion-transmitted infection, blood bank, demographic, hepatitis, Saudi Arabia

## Abstract

**Objectives:**

To establish a nationwide epidemiological profile of transfusion-transmittable infection (TTI) markers among seemingly healthy blood donors to update policies required to ensure blood safety.

**Methods:**

A nationwide, cross-sectional study was designed to examine donor demographics and TTI prevalence during 2020 using data provided by the Ministry of Health, Saudi Arabia.

**Results:**

Collectively, a total of 375,218 whole blood units were donated, of which 32,758 (8.7%) were excluded due to TTI-related risk. The exclusion was based on a positive nucleic acid amplification test (NAT) or seroreactivity to hepatitis B virus (HBV), hepatitis C virus (HCV), human immunodeficiency virus (HIV), human T-cell lymphotropic virus (HTLV-I/II), syphilis, or malaria. Notably, the central (37.6%) and southern (33%) regions were the epicenters of TTI-reactive blood donors. Hepatitis B virus markers accounted for 85.7% and were the overall most prevalent of TTI-positive donations, followed by HCV at 5.9% and syphilis at 5.6%. In particular, anti-HBc and HBsAg were most prevalent in the south, while HBV NAT was highest in the center.

**Conclusion:**

Hepatitis B virus, HCV, and syphilis carry the greatest risk of TTI in Saudi Arabia. Including HBsAg screening is a necessary precautious measure to maintain blood safety.

**B**lood transfusion plays a pivotal role in the management of hemorrhage and other hematological diseases.^1,2^ In Saudi Arabia, the healthcare system is overseen by the Ministry of Health (MOH), which has authority over the 13 national provinces. Besides formulating healthcare policies, the MOH has been providing free healthcare services through governmental hospitals and blood donation centers since its inception.^3^ All blood donors undergo screening for hepatitis B surface antigen (HBsAg), hepatitis B core antibody (anti-HBc), hepatitis C virus anti-body (anti-HCV), human immunodeficiency virus antibodies (anti-HIV-1/-2), human T-cell lymphotropic virus-I and -II antibodies (anti-HTLV I/II), syphilis, and malaria. In addition, nucleic acid testing (NAT) is also performed for hepatitis B virus (HBV), HCV, and HIV-1/-2 on all blood donations to curtail the risk of infection transmission.

Approximately, an average of 325,847 whole blood units are annually collected from 128 governmental blood banks distributed among the Kingdom’s 13 province.^4^ Thanks to established quality blood screening protocols, the global and local residual risk of transfusion-transmissible infections (TTIs) has been reduced over tim.^5^ However, number of challenges have been recognized by the National Transformation Plan (NTP) of Vision 2030 including population growth, increased financial burden, and inconsistent statistical reports submitted by the fragmented administration of MOH’s healthcare institutes. ^6,7^ To tackle these concerns within the context of transfusion medicine, mitigation strategies to curtail TTIs are often influenced by local epidemiology and cost-effectiveness considerations.^8^ In addition, accurate statistical reporting can offer the chance of investigating the prevalence of TTI rates in the asymptomatic infectious phase of seemingly healthy blood donors who pose the greatest risk to blood safety.

Available literature on TTI prevalence in Saudi Arabia is limited to regional or single-center studies, hemodialysis patients who underwent frequent blood transfusions, or to at-risk populations (such as heroin addicts or HIV-positive patients) where the prevalence of TTI is expected to be higher.^9-13^ In this report, we carried out a nationwide, cross-sectional study to analyze the frequency of TTI markers among seemingly asymptomatic blood donors in Saudi Arabia in 2020.

## Methods

This retrospective cross-sectional study included all blood donations performed in Saudi Arabia during the year 2020. Demographic data and TTI-reactivity were retrieved and analyzed from the MOH annual statistics report. Based on national blood donation and screening protocols, eligible donors must be between 18-60 years of age with no history or current of TTI disease, weigh >50 kg, with a hemoglobin level of >12.5 g/dL, and with normal blood pressure. All donations were screened for HBsAg, anti-HBc, anti-HCV, anti-HIV-1 and -2, anti-HTLV I/II, syphilis, and malaria by chemiluminescence and NAT. Unscreened whole blood units are initially quarantined until all blood units are screened. Confirmed TTI-positive cases are automatically registered in the Health Electronic Surveillance Network (HESN) for further management and are banned from blood donation.

This study was approved by the Ethical Committee of the MOH (21-71 E). All blood donors were requested to give informed consent prior to donation as per MOH regulations. The annual statistics report for the year 2020 was retrieved from the Blood Transfusion Services overseen by the MOH. Raw numbers of TTI reactivity were anonymously obtained without donors’ identification.

It was difficult to track all screening kits and equipment used over the study period; however, all were Food and Drugs Administration-approved as required by the MOH.^14^ For HBV and HCV serological screening, trademarks Architect i1000SR or Architect i2000 analyzers (Abbott, Abbott Park, IL, USA) operated by Abbott AxSym system with the ARCHITECT HBsAg Qualitative II Reagent kit, the ARCHITECT anti-HBs reagent kit, the ARCHITECT anti-HBc II Reagent kit, and the ARCHITECT anti-HCV Reagent were used. Readings >1.00 were labeled reactive.

For anti-HTLV-I/II screening the Abbott-Murex HTLV I/II enzyme immunoassay (EIA) (Murex Diagnostics/or/Abbott Gmbh, Delkenheim, Germany), latex immunoassay (LIA; INNO-LIA HTLV I/II score, FujiRebio, Japan), or chemiluminescent microparticle immunoassay (CMIA) Abbott ARCHITECT i2000SR were used. To confirm the initial screening results, positive samples were further tested by western blot (Autoblot 3000, Bio-Rad, Hercules, CA, USA). The Architect system HIV antigen/antibody combo CMIA was used for the detection of HIV p24 antigen and HIV-1/-2 antibodies. Human immunodeficiency virus Ab-Ag positive results were confirmed by INNO-LIA HIV I/II score. In addition, all donations were subject to NAT using CobasTaqScreenMPX Test, version 2.0 (Roche, Basel, Switzerland) for the simultaneous detection of HBV-DNA and RNA of HCV and HIV.

For syphilis screening, the CMIA Siemens immulite® 2000 XPi Immunoassay System was used to detect anti-Treponema pallidum (TP) antibodies, and the rapid RPR-carbon test and the Syphilis TP reagent Kit for the ARCHITECT i1000SR analyzer (Cypress diagnostics, Belgium) were used to confirm positive findings. For malaria, a thick blood smear stained with Giemsa stain and EIA (Evolis SystemsTM, USA) tests were performed.

### Statistical analysis

Transfusion-transmissible infections frequency rate was estimated according to the formula:^15^

TTI incidence=(confirmed cases)/(total number of annual donors) x 100,000

The nationwide prevalence of confirmed TTI markers was expressed in percentages relative to total TTIs using GraphPad Prism 9.0 (GraphPad Software, Inc., La Jolla, CA, USA). Association between TTI markers and geographical regions was evaluated using χ^2^ independence test followed by post-hoc pairwise Fisher exact test due to low expected frequencies. The graph was illustrated in a mosaic plot using HSV shading with user-defined cutoffs (2 and 4) based on heuristics to display the absolute value of the residuals associated with each tile.^16^ Correspondence analysis was performed to explore the relationships between each geographical region and each TTI marker frequency by cross-tabulation with total variance of 98.8%. The contingency table was graphically illustrated as a biplot. Mosaic plot and biplot were generated by factoextra R package version 4.1.2. (R Foundation for Statistical Computing, Vienna, Austria).

## Results

### Nationwide seroprevalence of TTI

A total of 375,218 donations were registered nationwide, of which 32,785 (8.7%) were found to be reactive to TTI biomarkers by routine chemiluminescence or NAT screening assays. Subsequent confirmatory tests performed on reactive donors revealed a total of 8,466 confirmed cases (Appendix 1). Repeated donation only by TTI-negative individuals may have contributed to the reported figures in this study. We have no data regarding the extent or magnitude of individual donation frequency in our cohort but we speculate that the effect is minimal given the large sample size.

The nationwide TTI prevalence is estimated to be 2,256 per 100,000 blood donors and the prevalence of TTI-positive donors per infectious agent is illustrated in Appendix 1. The most prevalent biomarkers among all confirmed serology and NAT among TTI-positive blood donors were HBV-related markers with anti-HBc (52.5%), HBV NAT (22.8%), and HBsAg (10.4%) positivity rates (Appendix 2). This was followed by syphilis (5.5%), anti-HCV (4.6%), anti-HTLV I/II (1.6%), and anti-HIV-1/-2 (0.8%).

### Regional distribution of TTI-reactive donors

The central region is the most populated region in Saudi Arabia and accounts for 15.1% of the national blood pool supply.^4^ It is therefore not surprising for the central region to have the highest TTI prevalence among blood donors with 3,188 cases representing 4.2% of regional donations, as shown in Table 1. The same region also had 37.7% of all TTI-positive cases (Table 1). To a lesser extent, the prevalence of TTI among blood donors was relatively high in the Southern region (2,798 cases which represented 3% of regional donations) (Table 1). Transfusion-transmissible infections-positive donors represented 1.1%-1.3% of total donations in the remaining regions. Correspondence analysis demonstrated that the central region is mostly associated with HBV NAT and, to a lesser extent, with syphilis and HTLV-I/II. The Western and Eastern regions are mostly associated with anti-HBc, whereas the Northern and Southern regions were mostly associated with HBsAg (Figure 2).

**Table 1:** - Prevalence (%)* of confirmed transfusion-transmissible infections (TTI) markers stratified by geographical region.

Region	HBV NAT	Anti-HBc	HBsAg	HCV NAT	Anti-HCV	HIV NAT	Anti-HIV1/2	Anti-HTLV I/II	Syphilis	Malaria	Total donors	Total Confirmed TTI (%)	Percentage TTI of regional donation
Northern	13 (0.03)	333 (0.9)	109 (0.3)	6 (0.01)	11 (0.02)	3 (0.01)	4 (0.01)	1 (0.003)	22 (0.1)	3 (0.008)	38,814	505 (6.0)	1.3
Central	1,464 (1.9)	934 (1.2)	109 (0.1)	51 (0.1)	209 (0.3)	18 (0.02)	32 (0.04)	79 (0.1)	292 (0.4)	0	76,044	3,188 (37.7)	4.2
Eastern	34 (0.1)	463 (1.1)	34 (0.1)	7 (0.01)	9 (0.02)	1 (0.002)	1 (0.002)	3 (0.01)	10 (0.02)	0	42,697	562 (6.6)	1.3
Western	107 (0.1)	1,064 (0.8)	157 (0.1)	10 (0.01)	36 (0.02)	5 (0.004)	9 (0.01)	2 (0.002)	29 (0.02)	4 (0.003)	126,307	1,413 (16.7)	1.1
Southern	313 (0.3)	1,649 (1.8)	471 (0.5)	31 (0.03)	132 (0.1)	12 (0.01)	25 (0.02)	46 (0.1)	118 (0.1)	1 (0.001)	91,356	2,798 (33.0)	3.1
Total	1,931	4,443	880	105	390	39	71	131	471	8	375,218	8,466 (100)	

Values are presented as numbers and percentages (%). *Percentage of TTI positive donors relative to total donors in the respective regions. HBsAg: hepatitis B surface antigen, HBc: hepatitis B core antibody, HIV: human immunodeficiency virus antibodies, HCV: hepatitis C virus antibody, HTLV I/II: human T-cell lymphotropic virus-I and -II antibodies, NAT: nucleic acid testing, HBV: hepatitis B virus

### Hepatitis B virus prevalence

**Figure 1:**
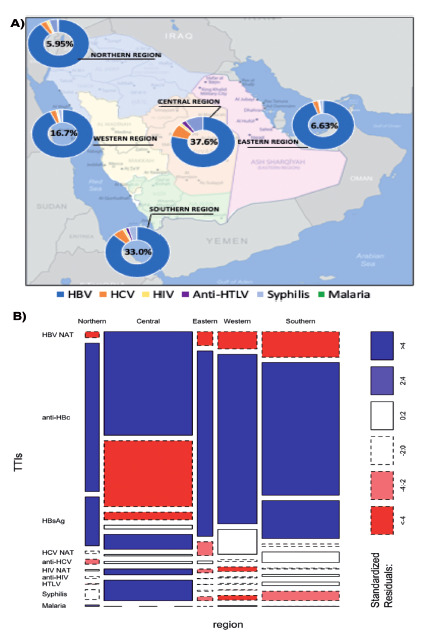
- Distribution of transfusion-transmittable infection (TTI) markers among Saudi geographical regions. **A**) Map of Saudi Arabia demonstrating the prevalence of TTI-positive markers to total confirmed cases in each geographical area. **B**) Mosaic plot illustrating the relationship between TTI markers and Saudi geographical regions. The width of the column is proportional to observations in geographical regions plotted on the horizontal axis. The vertical length of the bars is proportional to the number of confirmed TTI markers plotted on the vertical axis. The plot demonstrates Pearson residuals directly (cut-off values ±2 and ±4, significant at α=0.05 and α=0.0001), highlighting which tiles had more (blue) or less (red) observations than expected if the data was random (χ^2^ =2430.3, *p*<2.2 x 10-16). NAT: nucleic acid testing, HBV: hepatitis B virus, HBsAg: hepatitis B surface antigen, HBc: hepatitis B core antibody, HCV: hepatitis C virus anti-body, HIV: human immunodeficiency virus antibodies, HTLV: human T-cell lymphotropic virus

Stratification of TTI prevalence against the 5 main geographical areas demonstrated that anti-HBc seropositivity was the dominant TTI biomarker in all but the central region, which instead had the highest HBV NAT prevalence (Table 1 & Figure 1B). Anti-HBc is indicative of chronic or resolved infection and its prevalence rate ranged from 0.8% in the Northern and Western regions to 1.8% in the Southern region (Table 1). The prevalence of HBV markers at the provincial level is illustrated in Table 2. Alqounfoda, located in the Western region, recorded the highest prevalence of anti-HBc (5.2%), followed by Albaha (4.9%) and Aseer (4.8%) in the South (Table 2). The prevalence of anti-HBc was also relatively high in other provinces located in other regions such as Taif (3.9%), Beesha and Ahsa (3%), and Qassim (3%). The prevalence of HBsAg was highest in the Southern (0.5%) and Northern regions (0.3%) (Table 1). Further analysis of HBsAg prevalence revealed that Aseer stood out as the hyperendemic South-ern province with 1.2% of potential blood donors being carriers of HBsAg (Table 2). Similarly, Tabouk (0.5%) and the Northern border (0.4%) provinces had the highest HBsAg prevalence among other Northern region provinces.

**Table 2:** - Prevalence (%)* of HBV serology and NAT-positive blood donors among the 13 provinces.

Region	HBV NAT	Anti-HBc	HBsAg
***Northern region***			
Hail	0.05	2.11	0.05
Jouf	0.00	0.07	0.10
Tabouk	0.00	0.00	0.49
Northern	0.08	0.39	0.44
Alqurayat	0.07	2.48	0.14
***Central region***			
Riyadh	2.60	0.64	0.17
Qassim	0.00	2.90	0.07
***Eastern region***			
Eastern area	0.08	0.00	0.08
Hafr Albatin	0.09	0.04	0.09
Ahsa	0.08	2.95	0.08
***Western region***			
Mekka	0.00	0.00	0.00
Madinah	0.19	0.00	0.25
Alqounfoda	0.17	5.22	0.23
Jeddah	0.00	0.03	0.09
Taif	0.22	3.85	0.19
***Southern region***			
Aseer	0.31	4.81	1.19
Jazan	0.37	0.00	0.41
Najran	0.40	0.00	0.00
Albaha	0.34	4.89	0.33
Beesha	0.12	2.95	0.12

*Percentage of HBV-positive donors relative to total donors in the respective provinces. NAT: nucleic acid testing, HBV: hepatitis B virus, HBsAg: hepatitis B surface antigen, HBc: hepatitis B core antibody

Interestingly, 76% of nationwide HBV NAT-positive donors were reported in Riyadh (Appendix 3). Qassim did not report any HBV NAT-positive cases for the year 2020. Najran and Jazan reported the highest prevalence of HBV NAT-positivity in the Southern region at 0.4% each (Table 2). Positive HBV NAT is indicative of either acute, occult, or chronic infection depending on the presence of other serology markers. The lack of detailed NAT and seroreactivity profiles per donor limited our ability to accurately investigate the prevalence of the different phases of HBV infection and the prevalence of co-infections.

### Hepatitis C virus prevalence

Hepatitis C virus markers (anti-HCV and HCV NAT) were most prevalent in the central and Southern regions (Table 1 & Figure 1). Most anti-HCV positive cases were reported in Riyadh (0.4%), Aseer (0.3%), and Albaha (0.2%) (Table 3). The prevalence of HCV NAT was considerably low, with the highest prevalence recorded in the central region (0.1%), followed by the Southern region (0.03%), as shown in Table 1, with marked variation among provinces (Table 3).

**Table 3:** - Prevalence (%)* of HCV serology and NAT-positive blood donors among the 13 provinces.

Region	HCV NAT	Anti-HCV
***Northern region***		
Hail	0.03	0.03
Jouf	0.00	0.03
Tabouk	0.05	0.00
Northern	0.01	0.01
Alqurayat	0.00	0.00
***Central region***		
Riyadh	0.35	0.06
Qassim	0.06	0.06
***Eastern region***		
Eastern area	0.02	0.02
Hafr Albatin	0.02	0.00
Ahsa	0.01	0.01
***Western region***		
Mekka	0.00	0.00
Madinah	0.06	0.01
Alqounfoda	0.02	0.02
Jeddah	0.00	0.00
Taif	0.03	0.02
***Southern region***		
Aseer	0.25	0.02
Jazan	0.12	0.04
Najran	0.00	0.01
Albaha	0.24	0.03
Beesha	0.05	0.00

*Percentage of HCV-positive donors relative to total donors in the respective provinces. NAT: nucleic acid testing, HBV: hepatitis B virus, HBsAg: hepatitis B surface antigen, HCV: hepatitis C virus antibody

### Prevalence of other TTI markers

Discarded blood units due to HIV, HTLV, syphilis, or malaria reactivity were predominantly reported in the central and Southern regions. The prevalence of anti-HIV was 0.041% in the central region and 0.027% in the Southern region (Table 1). Prevalence in other regions ranged from 0.002% to 0.009% (Table 1). A similar pattern was observed for HIV NAT and anti-HTLV-I/II, which were most prevalent in the central and Southern regions (Appendix 4). Only 8 cases of malaria were reported nationwide; 4 in the West, 3 in the North, and 1 in the South.

## Discussion

The prevalence of TTI-reactivity among blood donors in Saudi Arabia during 2020 is reported in this study. Blood donors with confirmed TTI reactivity represented 8.7% of nation-wide asymptomatic donors, mostly reported from the center (37%) and the South (33%), thus urging the need to investigate the reasons for the increased TTI reactivity in those regions. This rate of donor deferral due to TTI reactivity is lower than that of other Eastern Mediterranean countries.^17,18^ The WHO global status report on blood safety and availability, published in 2013, demonstrated that the median rate of global blood donor deferral was 12%, and that of Saudi Arabia was 6.5%.^19^ However, the reported rates did not clarify whether donor deferral was solely due to TTI reactivity or to absence of donor selection criteria.

Donors with confirmed reactivity to HBV biomarkers comprised 85.6% of all confirmed TTI cases. This is not surprising given that Saudi Arabia has a low to moderate prevalence of HBV chronic disease (such as ≥2% of population).^20^ Anti-HBc represented 52.5% of all TTI-positive markers, followed by HBV NAT (22.8%) and HBsAg (10.4%). These distributions are in agreement with local regional studies.^21^ Anti-HBc is a valuable marker for HBV exposure irrespective of the current infectious state, especially at the end of a resolving infection when HBsAg and HBV NAT may not be positive. Hepatitis B surface antigen is indicative of active infection whereas HBV NAT is a marker of chronic infection. The relatively high frequencies of HBsAg and HBV NAT among blood donors is an interesting observation given that HBV vaccination has been mandatory in Saudi Arabia since 1989.^22^ In addition, the country follows stringent screening programs for all pilgrims and non-Saudi employees. In our study, donor ages and vaccination profiles were not included in the annual statistics report; thus, we were not able to stratify HBV seroprevalence based on age groups or explore whether a booster vaccine is recommended for isolated anti-HBc vaccinated subjects. It has been reported that even after HBV vaccination, the prevalence of anti-HBs (such as a marker of immunization that was not reported here) can progressively decrease with age in the presence or absence of anti-HBc.^23^

An earlier, population-based survey in Saudi Arabia identified the Southern region and Tabouk province as hyperendemic areas for HBV.^24^ It was hypothesized that HBV prevalence in these regions might be influenced by populations of neighboring countries known to be HBV endemic. Three decades later, our study demonstrated a different HBV prevalence pattern with the Southern and central regions emerging as areas with the highest HBV prevalence.

Every country must establish its own screening strategy based on its unique epidemiology and economy. For instance, in Germany, mandatory HBV screening includes HBsAg and anti-HBc, while HBV NAT is optional and only considered on a case-by-case basis.^25^ Such a strategy was based on periodic epidemiological studies confirming the low endemic state of HBV in Germany.^25^ Hepatitis B surface antigen screening is considered of minimal value in reducing HBV-associated risk when performed in combination with HBV NAT and anti-HBc tests.^26^ Whether HBsAg screening is crucial for blood safety was not analyzed here. This is attributed to the nature of the retrieved data which provided the sum of TTI-reactive donors per province without detailing each donor’s serology and NAT profile. Given that Saudi Arabia is considered a high-income country and is a destination for working force from all around the world, the inclusion of HBsAg test in the screening protocol may be justified as an affordable precautious measure. Additionally, the possible transmission of occult HBV from NAT-negative donors and the low-moderate HBV endemicity similarly justify the inclusion of HBsAg in the routine screening panel.^27,28^

The prevalence of HCV markers was relatively low among blood donors. This finding was expected provided that the Saudi Arbaia has a very low hepatitis C viremic prevalence (0%-0.6%).^29^ Likewise, the prevalence of other TTIs was considered very low and within the range of upper-middle income countries.^19^

### Study limitation

It is important to stress that the nature of our data merits cautious interpretation and limits the generalizability of our findings. A major limitation of this study was that the sum of TTI-reactive donors in each province was reported instead of detailed serology and NAT profile per donor. This did not allow for further investigation of disease epidemiology among blood donors or for assessment of variance between screening and confirmation.

Our findings strongly support the health sector’s NTP initiative, which proposes establishing a centralized blood bank management model with interconnected systems. This is critical as the current fragmented system resulted in inconsistent data reporting, inability to determine blood bank shortage or overload, and which departments are associated with the most waste or inappropriate blood usage. Thus, a centralized management system with a database where blood donor information could be integrated would allow for continuous and accurate TTI surveillance and provide support to preventive plans and risk management for emerging viruses (such as Zika or Dengue viruses). The initiative would also improve financial management by setting up a single national inventory to accommodate supply and demand and to establish comprehensive metrics for quality performance.

In conclusion, the current study demonstrates that reactivity to HBV dominated other TTI markers on a national level, with the central and Southern regions being hyperendemic for HBV. Until a detailed public epidemiology profile is available, the inclusion of HBsAg test in the current screening strategy seems to be a necessary precautious measure to ensure the highest standards of blood safety. However, a consensus is required to determine the clinical utility and financial feasibility of routinely testing for HBsAg. Such a consensus could be achieved through an integrated, nationwide network with a single inventory.

We also recommend that future MOH statistical reports provide detailed donor demographics and essential variables (such as age, gender, BMI, socioeconomic status, medical history, medication intake, and donation frequency) and report TTI pro-files per donor for more efficient data analysis.

## Appendix

**Appendix 1:** - Prevalence (%)* of screened and confirmed transfusion-transmittable infection (TTI) markers among 375,218 potential blood donors in Saudi Arabia, 2020.

Serological parameter	Number of reactive blood donors (%)*		Prevalence of confirmed TTI per 100,000 donors
	Screened	Confirmed	
HBsAg	3,460 (0.92)	880 (0.23)	234.5
Anti-HBc	20,550 (5.47)	4,443 (1.18)	1,184.1
Anti-HCV	1,376 (0.36)	390 (0.10)	103.9
Anti-HIV-1/2	712 (0.19)	68 (0.01)	18.1
Anti-HTLV I/II	644 (0.17)	131 (0.03)	34.9
Syphilis	1,500 (0.40)	471 (0.12)	125.5
Malaria	1,825 (0.48)	8 (0.002)	2.1
Total	30,067	6,391	
***Molecular testing***			
HBV NAT	2,424 (0.65)	1,931 (0.51)	514.6
HCV NAT	178 (0.04)	105 (0.02)	27.9
HIV NAT	89 (0.02)	39 (0.01)	10.3
Total	2,691	2,075	
		

*Percentage of total donors.

**Appendix 2:** - Number of cases (n) and frequencies (%) of ransfusion-transmittable infection markers distributed among donors with confirmed reactivity.

Serological parameter	n	%
HBsAg	880	10.4
Anti-HBc	4,443	52.5
Anti-HCV	390	4.6
Anti-HIV 1/2	68	0.8
Anti-HTLV I/II	131	1.5
Syphilis	471	5.5
Malaria	8	0.09
***Molecular testing***		
HBV NAT	1,931	22.8
HCV NAT	105	1.2
HIV NAT	39	0.4
Total	2,075	100

Values are presented as number and percentage (%). HBsAg: hepatitis B surface antigen, HBc: hepatitis B core antibody, HIV: human immunodeficiency virus antibodies, HCV: hepatitis C virus antibody, HTLV I/II: human T-cell lymphotropic virus-I and -II antibodies, NAT: nucleic acid testing, HBV: hepatitis B virus

**Appendix 3:** - Nationwide frequencies of confirmed TTI markers distributed against geographical region expressed as a percentage of the total TTI frequency.

	HBV NAT	Anti-HBc	HBsAg	HCV NAT	Anti-HCV	HIV NAT	Anti-HIV 1/2	Anti-HTLV I/II	Syphilis	Malaria
Northern	1	7	12	6	3	8	6	1	5	38
Central	76	21	12	49	54	46	45	60	62	0
Eastern	2	10	4	7	2	3	1	2	2	0
Western	6	24	18	10	9	13	13	2	6	50
Southern	16	37	54	30	34	31	35	35	25	13
Total	100	100	100	100	100	100	100	100	100	100

Values are presented as percentage (%). HBsAg: hepatitis B surface antigen, HBc: hepatitis B core antibody, HIV: human immunodeficiency virus antibodies, HCV: hepatitis C virus antibody, HTLV I/II: human T-cell lymphotropic virus-I and -II antibodies, NAT: nucleic acid testing, BV: hepatitis B virus, TTI: ransfusion-transmittable infection

**Appendix 4:** - Prevalence (%)* of other TTI markers in blood donors among the 13 provinces.

Regions	HIV NAT	Anti-HIV 1/2	Anti-HTLV I/II	Syphilis
***Northern region***				
Hail	0.02	0.02	0	0.09
Jouf	0	0	0	0.13
Tabouk	0	0.007	0	0
Northern	0.1	0.01	0.01	0.08
Alqurayat	0	0%	0	0
***Central region***				
Riyadh	0.02	0.04	0.13	0.5
Qassim	0.01	0.03	0.01	0.02
***Eastern region***				
Eastern area	0	0	0	0.01
Hafr Albatin	0	0	0.04	0.01
Ahsa	0.006	0.006	0	0.03
***Western region***				
Mekka	0	0	0	0
Madinah	0.01	0.009	0.003	0.02
Alqounfoda	0	0	0	0.11
Jeddah	0	0.005	0.002	0
Taif	0.007	0.007	0%	0.06
***Southern region***				
Aseer	0.01	0.08	0.18	0.4
Jazan	0.01	0.01	0.002	0.02
Najran	0.01	0	0	0
Albaha	0.01	0.01	0	0.06
Beesha	0	0	0.03	0.05

*Percentage of other TTI-positive donors relative to total donors in respective provinces.

TTI: ransfusion-transmittable infection, HIV: human immunodeficiency virus antibodies,

NAT: nucleic acid testing, HTLV I/II: human T-cell lymphotropic virus-I and -II antibodies
